# Atypical presentation of cardiomyopathy in a case of maternal mortality that was demonstrated as hypovolemic shock

**DOI:** 10.1002/ccr3.5010

**Published:** 2021-10-25

**Authors:** Banfsheh Mashak, Maryam Hashemnejad, Maliheh Fakehi, Zohreh Heidary, Roghayyeh Mirmajidi, Marjan Ghaemi

**Affiliations:** ^1^ Kamali Hospital School of Medicine Alborz University of Medical Sciences Karaj Iran; ^2^ Department of Gynecology and Obstetrics Shahid Akbarabadi Hospital Iran University of Medical Sciences Tehran Iran; ^3^ Vali‐e‐Asr Reproductive Health Research Center Tehran University of Medical Sciences Tehran Iran

**Keywords:** cardiomyopathy, COVID‐19, hypovolemic shock, maternal mortality, post‐partum hemorrhage

## Abstract

Periconceptional and prenatal care should be continued even during COVID‐19 pandemics. Indeed, prevention and intervention programs for managing heart failure with aggressive resuscitation and invasive monitoring help to provide the best outcomes in cardiomyopathies. PPH may be associated with cardiac diseases and the resuscitation measures need modification to prevent maternal mortality.

## Graphical abstract

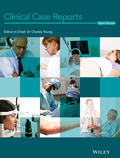

## 1 BACKGROUND

A 21‐year‐old woman gravid 1 with poor prenatal care manifested cardiovascular collapse after vaginal delivery. With suspicion of postpartum hemorrhage, volume replacement therapy was initiated. Shortly after, she developed pulmonary edema, respiratory distress, and cardiac failure and could not survive after a series of resuscitative efforts. On autopsy, cardiomyopathy with diffuse inflammatory cell infiltration was detected.

Cardiac disease is a leading cause of maternal and fetal morbidity and mortality in pregnancy.^1^ However, it is the commonest cause of maternal deaths in developed countries,^2^ but it is difficult to assess in developing countries due limited availability of mortality reports. In most women,^3^ cardiac diseases would be symptomatic in the third trimester and puerperium,^4^ with increased risks of fetal growth restriction and stillbirth, preterm delivery, and postpartum hemorrhage.^5^

Accurate care in all women before and after delivery is mandatory. It allows early recognition of the woman who is becoming critically unstable.^6^ Likewise, maternal near miss leading to maternal death due to unrecognized cardiac condition. On the other hand, postpartum hemorrhage (PPH) may be a causes of sudden maternal collapse. Therefore, knowing the physiological differences of pregnant women is very essential for proper resuscitation.^6^

The lack of awareness among clinicians and inadequate perinatal care may lead to prolonged and persistent cardiac dysfunction in the reproductive age mother and prolonging the hospital stay. Proper diagnosis and prompt treatment by the healthcare team not only save the mother and fetus life but also decline the economic burden of the disease.^7^ Here, we report a case of maternal mortality with cardiac shock due to cardiomyopathy shortly after delivery that was misdiagnosed as a hypovolemic shock.

## 2 CASE PRESENTATION

A 21‐year‐old, gravid 1 woman with term pregnancy attended an academic hospital with labor pain. She had two prenatal care visits in the first and second trimester with normal appearance and did not have any appointment with obstetricians in the third trimester due to COVID‐19 pandemics. She experienced mild episodes of dyspnea during the third trimester of pregnancy that did not consider important for her to seek consultation. Her pulse rate was 88 beats per minute with a respiratory rate of 14 per minute, blood pressure 132/88 mmHg, and oral temperature 37.5° centigrade. She had no dyspnea. In cardiovascular exam, the heart sound was normal and the lung was clear. The O2 saturation was 97%. On vaginal examination, cervical dilatation was 3 cm with effacement 50%; cephalic presentation, and intact amniotic membrane. The fetal heart rate was 140 beats per minute. The patient was admitted to the labor unit due to the regular uterine contractions. In general appearance, she looked good with no apparent distress.

According to the patient's biography, this was her first pregnancy. She denied a history of any disease or surgery except a subtle common cold about 2 weeks ago. Her family history was non‐significant. She did not take any medications other than iron and multivitamin supplements. Labor progressed without any problems and after about 8 h she delivered a full‑term girl with the Apgar score of 9 and 10 in the first and the fifth minute, respectively.

The third stage of the labor between deliveries of the newborn until the placenta last about 10 min without abnormal hemorrhage. For PPH prophylaxis 30 IU of IV Oxytocin in 500 ml NaCl 0.9% was administered in this stage. After delivering the placenta, she experienced an onset of uterine atonia and postpartum hemorrhage (about 700 ml) that was controlled with intravenous oxytocin and uterine massage. After the second episode of bleeding (about 550 ml) in 15 min, because of deteriorating hemodynamics despite intravenous fluid, she was transferred to the operating room for invasive resuscitation and uterine and vaginal examination under anesthesia to investigate the potential source of bleeding. The total estimated blood loss was 1200 ml.

She was pale but alert with tachycardia of about 125 beats per minute with an O2 saturation of 93% and systolic blood pressure of 73/49 mm Hg. After 2 large bores peripheral lines, 2500 cc crystalloid fluids besides 500 ml of colloid were infused. Shortly after, cardiac monitoring in the operating room showed ventricular tachycardia at a rate of 180 beats per minute and then PSVT (Paroxysmal Supraventricular Tachycardia), which was controlled by injecting lidocaine with an initial dose of 100 mg intravenously and then 4 mg/min as the maintenance dose. After that, the heart rhythm was converted to sinus tachycardia at a rate of 120 beats per minute and the patient's blood pressure reaches 110/60. Two pack cells (cross match) were transfused by jugular vein.

After being stable, the physical examination by two obstetricians in the operation room revealed no evidence of uterine atonia, laceration, or detectable hematoma. The hemorrhage was stopped with a contracted and global uterus. Abdominal and pelvic ultrasound was performed with no evidence of intra‐abdominal bleeding, but the patient was got deteriorated and progressed to pulmonary edema. Furosemide was administered with an initial dose of 300 mg and then 2 to 4 mg per hour as a maintenance dose. The patient's urinary output was 300 cc. A 12 lead electrocardiography showed ventricular tachycardia with a wide QRS complex. Cardiac echocardiography was performed by a cardiologist that showed ejection fraction about 10% to 15% and moderate mitral valve regurgitation.

After two cardiac resuscitations, she was intubated and fentanyl and midazolam were started as a drip, and the patient was transferred to the intensive care unit with an oxygen saturation rate of 95–98%. After three hours, she experienced a high‐grade fever (40.5° centigrade) that was resistant to intravenous Apotel. Therefore, broad‐spectrum antibiotics besides epinephrine drip started and COVID‐19 test by polymerase chain reaction (PCR) was performed for her with negative results.

**FIGURE 1:**
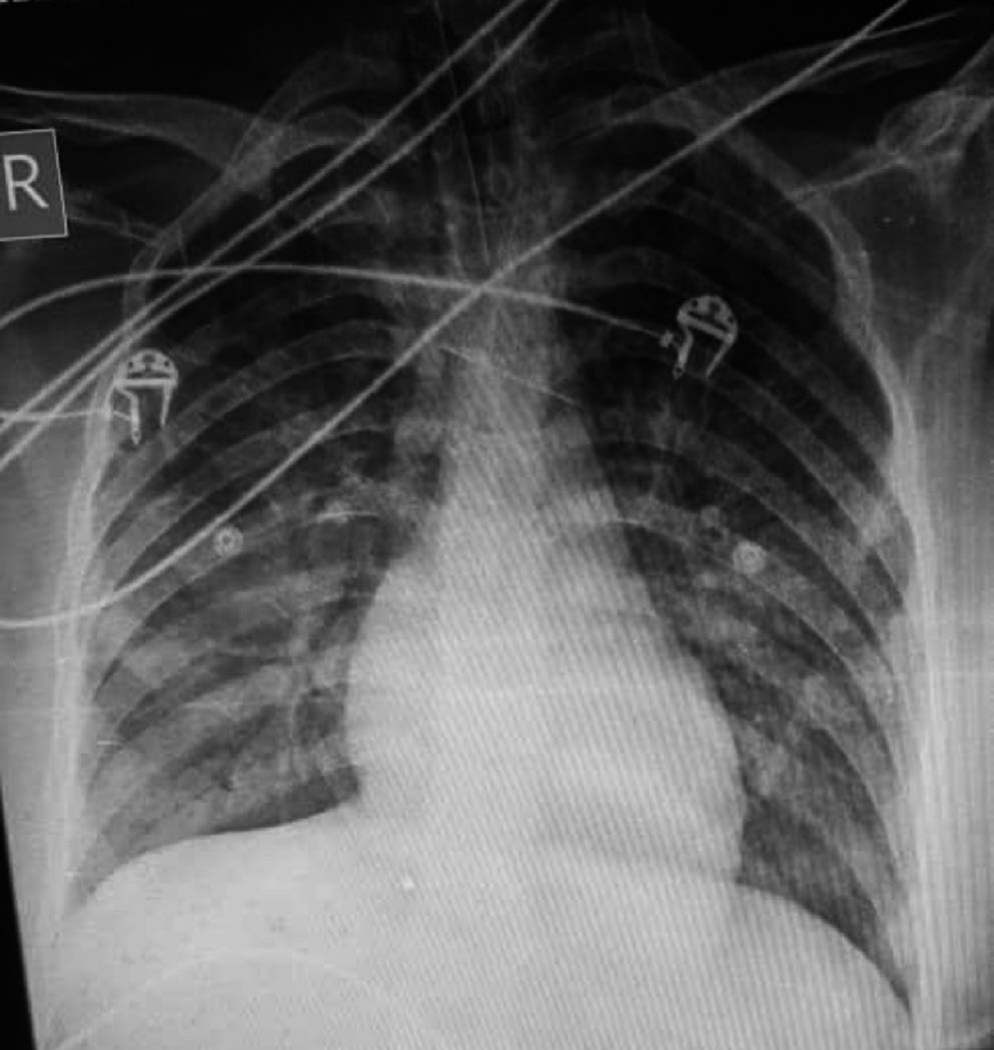
Chest X‐ray of the case that is shown bilateral patchy infiltration without cardiomegaly

Chest X‐ray showed bilateral patchy infiltration without cardiomegaly (Figure 1). Despite all supportive and therapeutic proceedings, the patient experienced ventricular tachycardia, which returned with cardiac shock and administration of epinephrine and atropine; and unfortunately, in the second attack, she deceased due to not responding to cardiac resuscitation 12 h after delivery. The autopsy revealed cardiomyopathy with diffuse inflammatory cell infiltration with a small heart size and fibrotic tissue as well as the aortic stenosis. Thin layers of pus in the bases of the lungs were observed either.

This paper was conducted according to the principles of the Helsinki Declaration. The principles of confidentiality were observed, and the identity of the individual was not disclosed.

## 3 DISCUSSION

It is believed that the rate of pregnancy‐related maternal mortality dropped in the last decades, but it is still unacceptably high.^8^ Cardiac diseases, such as heart failure, remained as one of the leading causes of maternal mortality. Differentiating the vague symptoms of heart failure from common symptoms in the third trimester of pregnancy is challenging for obstetricians and cardiologists. About 60% of hospital admission due to peripartum heart failure occurs in the puerperium, and 5% of all postpartum admissions are associated with HF.^9^ Therefore, close monitoring of high‐risk postpartum women before discharging is mandatory. If the symptoms are uncertain, it is crucial to consider cardiomyopathy. Our patient also reported shortness of breath for a while but did not go for consultation due to the COVID‐19 pandemic.

The differential diagnosis for postpartum collapse includes postpartum hemorrhage as most prevalent, thromboembolic disease, amniotic fluid embolism, sepsis, intracranial hemorrhage drug overdose and the cardiovascular reasons such as acute myocardial infarction, aortic dissection, and cardiomyopathy. Symptoms and signs indicative of these three cardiac causes include central chest or interscapular pain, a wide pulse pressure, and new cardiac murmur.

One of the differential diagnoses of this patient is viral myocarditis. As reported, myocardial damage is relatively common in COVID‐19 pandemics accounting for 7–23% with a higher incidence of morbidity and mortality.^10^ Due to her flu‐like symptoms in the last 2 weeks, persistent fever, and purulent discharge based on the lungs despite negative PCR, the infection with COVID‐19 and further myocarditis was suspected. The frequency of reporting cardiomyopathy due to COVID 19 in pregnancy may have a similar spike in the overall pregnancy‐related maternal mortality attributed to the 2009 influenza A (H1N1) pandemic.^11^ We evaluate the possibility of COVID‐19 infection due to the pandemics. But due to cardiac fibrosis, the acute condition was less common.

Women with congenital heart diseases are reported to have twofold higher chance for postpartum hemorrhage (PPH), but the etiology is not well understood.^9^ It is assumed that the higher CARPREG (Cardiac Disease in Pregnancy Study) scores are associated with increased PPH. This may be due to the physiological changes occurring during pregnancy or labor process reveal or exacerbate the symptoms.^12^ Considering that our case had normal single, term pregnancy and the autopsy result, postpartum hemorrhage could be a consequence of her heart disease.

Takotsubo cardiomyopathy may be another differential diagnosis. This stress‐induced cardiomyopathy is characterized by new‐onset left ventricular dysfunction and would be symptomatic close to delivery that makes it difficult to differentiate from peripartum cardiomyopathy.^13^ Electrocardiogram abnormalities include ST segment elevation in the anterior and precordial leads and elevated cardiac troponin and brain natriuretic peptide levels are seen in most cases.^14^ In this case, we did not have ST elevation or elevated cardiac enzymes.

Restrictive cardiomyopathy could be the front line in the differential diagnosis. It is complicated by increased myocardial rigidity. Increasing the plasma volume in pregnancy may lead to volume overload heart failure^15^; otherwise, they may be asymptomatic before pregnancy.^16^

Most cardiovascular complications develop during delivery or postpartum. Delivery and its complications such as hypovolemia secondary to blood loss may poorly tolerate in this population. The episodes of hemorrhage in our case may deteriorate her cardiac function that led to heart failure.

The principle of management of heart is intensive care unit (ICU) admission and close monitoring of the vital sign is the main step. When necessary, oxygen should be added by noninvasive ventilation to achieve arterial oxygen saturation above 95%. Other medical treatments such as diuretics, nitrate, and inotropic may be administered. Precise fluid handling is critical in these people because volume overload may lead to pulmonary edema as our patient.

For managing such patients neither central venous pressure monitoring nor pulmonary capillary wedge pressure could give an accurate measurement of left ventricular filling. Though implantable cardioverter defibrillator (ICD) implants have good outcomes in pregnant cases with heart disease.^17^ Some other modalities include medical, electrophysiological, percutaneous transluminal septal myocardial ablation, and surgical therapy that was proposed in another study,^18^ but unfortunately, we had not available invasive cardiac services in our center.

Cardiomyopathy in pregnancy is a serious condition and the prognosis widely depends on early diagnosis and treatment. Indeed, it is crucial to consider that women with cardiac diseases are at a higher risk for postpartum hemorrhage.^7^

## 4 CONCLUSION

Periconceptional and prenatal care should be provided for all women even during COVID‐19 pandemics. Consultation with a cardiologist may be mandatory in prenatal care. Indeed, prevention and intervention programs for managing heart failure with aggressive resuscitation and invasive monitoring help to provide the best outcomes in cardiomyopathies. As the diagnosis of cardiac condition was not made initially immediately after collapse, the resuscitative measures instituted have caused pulmonary edema and more strain on heart.

## CONFLICTS OF INTEREST

The authors declare that there are no conflicts of interests.

## AUTHOR CONTRIBUTIONS

M.G involved in project development and manuscript writing. B.M involved in data collection and management, and manuscript editing. M.H, M.F, R.M, Z.K.S, and Z.H involved in manuscript editing and data management. All authors have read and approved the manuscript.

## CONSENT

Written consent was signed upon admission by the patient included in this study to use their information in research studies and as available for review. No personal information had been published, and the identity of the participants had not transpired. The informed consent was obtained from her husband to publish the data and the image of her wife after her deceases.

## Data Availability

Data available on request from the authors (The data that support the findings of this study are available from the corresponding author upon reasonable request).
